# Mechanistic Insights into Lipooligourea-Lipid Membrane Interactions

**DOI:** 10.1021/acs.jpcb.5c02112

**Published:** 2025-06-21

**Authors:** Kinga Burdach, Zuzanna Reterska, Arkadiusz Grempka, Damian Dziubak, Joanna Juhaniewicz-Dębińska, Paulina Bachurska-Szpala, Karolina Pulka-Ziach, Sławomir Sęk

**Affiliations:** 1 Faculty of Chemistry, Biological and Chemical Research Centre, 49605 University of Warsaw, Żwirki i Wigury 101, Warsaw 02-089, Poland; 2 Faculty of Chemistry, 49605 University of Warsaw, Pasteura 1, Warsaw 02-093, Poland

## Abstract

Understanding how synthetic peptidomimetics interact with bacterial membranes is key to developing next-generation antimicrobials. In this study, we investigate the membrane-disruptive behavior of C10-OU4, a cationic lipooligourea foldamer that mimics the amphiphilic architecture of antimicrobial lipopeptides. Using a multitechnique approach—Langmuir monolayer analysis, quartz crystal microbalance with dissipation monitoring (QCM-D), and attenuated total reflection-Fourier transform infrared spectroscopy (ATR-FTIR)—we probe the concentration-dependent interactions of C10-OU4 with lipid membranes that model Gram-positive bacterial membranes. At low concentrations (1 μM), C10-OU4 adsorbs to the membrane surface, inducing minor structural perturbations limited to the polar headgroup region. Increasing the concentration to 5 μM results in significant acyl chain disorder, partial membrane solubilization, and likely, micelle-like aggregate formation, as evidenced by QCM-D frequency shifts and ATR-FTIR data. At 10 μM, near the minimal inhibitory concentration, membrane disintegration becomes extensive, with the lipooligourea adopting orientations suggestive of random or tilted insertion geometries. These findings support a multimodal mechanism of action that transitions from surface association to full bilayer disruption in a concentration-dependent manner. The combined use of structural and dynamic measurements provides detailed insight into the physicochemical principles underlying lipooligourea–membrane interactions, offering a foundation for the rational design of membrane-active foldamer antibiotics.

## Graphical abstract

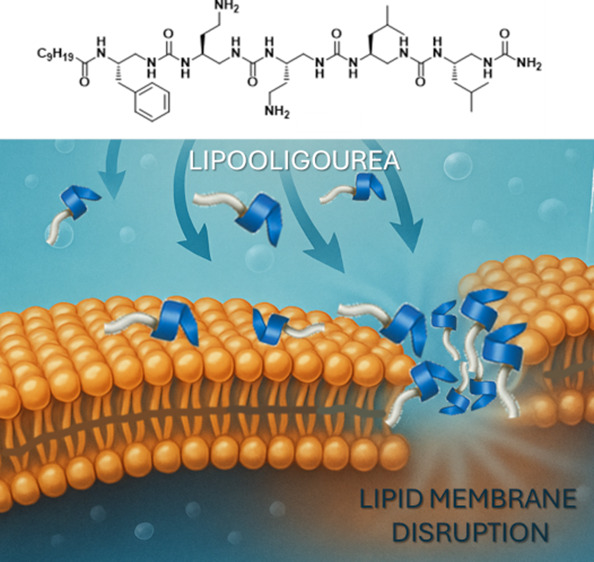

## Introduction

The rise of multidrug-resistant (MDR) bacterial infections has become one of the most pressing global health concerns, posing a significant challenge to modern medicine. The widespread and often excessive use of conventional antibiotics has accelerated the evolution of resistance mechanisms, rendering many existing treatments ineffective.^1^ As most antibiotics exert their effects through highly specific biochemical pathways—such as inhibition of protein synthesis, cell wall biosynthesis, or nucleic acid metabolism—bacteria can rapidly adapt through target modification, efflux pump overexpression, or enzymatic drug degradation.^1–3^ This has created an urgent demand for alternative antimicrobial strategies that are less susceptible to resistance development.

One promising avenue involves targeting the bacterial cell membrane, a universal and essential structure that is more difficult for pathogens to modify without compromising viability. Membrane-active agents, such as antimicrobial peptides (AMPs) and lipopeptides, often exert their effects through physical interactions with lipid bilayers, leading to permeabilization, depolarization, or even full membrane disruption.^4–13^ Due to their amphiphilic nature—comprising both hydrophobic and cationic regions—these compounds can selectively associate with negatively charged bacterial membranes, sparing mammalian cells that possess predominantly zwitterionic phospholipid compositions. Although several naturally occurring membrane-targeting antibiotics, such as polymyxins and daptomycin, have been clinically approved, they often suffer from limitations including poor stability, toxicity, and susceptibility to proteolytic degradation.^14^ To overcome these drawbacks, increasing attention has turned toward synthetic peptidomimetics, particularly foldamers, which offer tunable secondary structures, high proteolytic resistance, and modular design. Among these, oligourea-based foldamers have emerged as compelling candidates due to their ability to adopt well-defined 2.5-helical conformations that remain stable in aqueous environments.^15,16^ These helices can be decorated with cationic side chains to promote electrostatic interactions with bacterial membranes, and lipophilic acyl chains to enhance membrane insertion.^17,18^ This dual-interaction strategy mimics the natural amphiphilicity of antimicrobial lipopeptides but within a robust synthetic scaffold. Recent studies have demonstrated that such lipooligoureas exhibit antimicrobial activity—particularly against Gram-positive strains—and that their activity can be modulated by subtle changes in their sequence, charge distribution, and hydrophobicity.^19^ Despite their promise, the mechanistic basis for membrane disruption by lipooligoureas remains insufficiently understood. In particular, it is unclear how these molecules behave at different concentrations, how they interact with the polar headgroups versus the hydrophobic core of lipid membranes, and whether their mode of action involves membrane thinning, micelle formation, or disruption of lipid packing.

To address these questions, we investigated the interaction of a model lipooligourea, C10-OU4 (Scheme 1), with artificial lipid membranes mimicking the anionic composition of bacterial inner membranes, using a combination of complementary biophysical techniques. Antibacterial activity of C10-OU4 was recently reported by our group.^19^ By employing Langmuir monolayer isotherms, we characterized initial binding and insertion behavior at the air-buffer interface. Quartz crystal microbalance with dissipation (QCM-D) provided real-time insights into membrane perturbation, structural rearrangements, and mass redistribution in supported lipid bilayers. Finally, attenuated total reflection Fourier-transform infrared spectroscopy (ATR-FTIR) allowed us to probe lipid acyl chain ordering and molecular orientation at various lipooligourea concentrations.

**Scheme 1:**
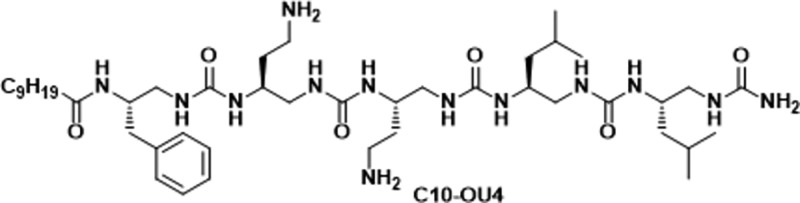
Structure of Lipooligourea C10-OU4

As our model membrane, we chose three-component mixture consisting of DPPG, POPG, and cardiolipin (CL) at 1:1:2 mol/mol/mol ratio. This composition is supposed to reflect the specific features of the *Staphylococcus aureus* membrane, which is rich in anionic lipids like phosphatidylglycerol (PG) and CL.^20^ Therefore, our lipid model stays within this range and reflects the natural negative charge of the bacterial membrane. In addition, previous studies show that cardiolipin is not only a structural component but plays an important functional role. For example, Koprivnjak et al. demonstrated that during transition to stationary phase, most PG in S. aureus is converted into CL, especially due to the action of the Cls2 enzyme.^21^ This accumulation is even stronger when bacteria are phagocytosed by neutrophils. Another important study by Jiang et al. showed that mutations in the cls2 gene, which increase CL synthesis, help S. aureus to survive daptomycin treatment and avoid immune response.^22^ These strains had significantly more CL and less PG in their membranes. Considering this, we believe that our lipid model with increased CL is justified and relevant for mimicking the S. aureus membrane under conditions of stress, antibiotic exposure or immune challenge. The same lipid mixture has been used in several studies from our group before, where it gave stable and reproducible bilayers for biophysical experiments.^9,23–25^

Our findings reveal a concentration-dependent, multimodal interaction of C10-OU4 with model lipid membranes, ranging from superficial binding at low concentrations to pronounced disruption and membrane disassembly at higher doses. These results not only shed light on the mode of action of synthetic lipooligoureas but also provide a conceptual framework for their rational design as next-generation antimicrobial agents targeting membrane integrity.

## Experimental Section

### Chemicals

The lipids 1-palmitoyl-2-oleoyl-*sn*-glycero-3-phosphoglycerol (POPG), 1,2-dipalmitoyl-*sn*-glycero-3-phosphoglycerol (DPPG), and 1’,3′-bis­[1,2-dimyristoyl-*sn*-glycero-3-phospho]-glycerol (CL) were obtained from Avanti Polar Lipids Inc. Ultrapure methanol and chloroform, used as lipid solvents, were sourced from Sigma-Aldrich. Avantor Performance Materials Poland S.A. supplied sodium chloride, sodium phosphate, potassium chloride, and potassium phosphate. Chempur supplied analytical-grade sodium hydroxide and hydrochloric acid, which were used to adjust the pH of the 0.01 M phosphate-buffered saline (PBS) solution to 7.4. Stock solutions of lipooligourea and PBS buffer were prepared using Milli-Q water with a final resistivity of 18.2 MΩ × cm.

### Synthesis of Lipooligourea C10-OU4

The synthesis of C10-OU4, a helical pentameric lipooligourea foldamer, was carried out via microwave-assisted solid-phase synthesis (at 50 W, 50 °C) on NovaPEG Rink Amide resin. The assembly involved sequential coupling of five custom-designed azido-functionalized carbamate building blocks (BB1–BB4) in the presence of DIPEA in DMF. Each coupling step was monitored by the chloranil test to ensure completion. Following oligourea chain assembly, azide-to-amine conversion was achieved by Staudinger reduction using 1 M PMe3 in THF in a dioxane/water mixture. Coupling and reduction steps were performed under microwave irradiation for 30 min. Each step was repeated once or more than once, if necessary, to be sure that the reactions were completed. The terminal hydrophobic tail (C9H19COOH) was introduced via amide bond formation using HBTU activation. Final cleavage from the resin and global deprotection were performed using a TFA-based cocktail (95:2.5:2.5 TFA: H2O:TIS), and the crude product was purified by semipreparative RP-HPLC. The counterion (TFA^–^) was exchanged for Cl^–^ using a Dowex ion-exchange resin. Detailed synthetic protocols, reagent conditions, and analytical characterization (HPLC, HRMS, NMR) are available in our previous work.^19^

### Surface Pressure Measurements

Lipid monolayers at the air/buffer interface were generated using a KSV NIMA Langmuir trough (Biolin Scientific, Sweden) equipped with two adjustable hydrophilic barriers. Surface pressure measurements were conducted using a Wilhelmy plate made of filter paper. Prior to each experiment, the trough and barriers were thoroughly cleaned with a chloroform/methanol mixture, followed by rinsing with Milli-Q water. Lipooligourea C10-OU4 was dissolved in water, while lipid solutions were prepared as follows: POPG was dissolved in chloroform, DPPG in a chloroform/methanol mixture (65:35, v/v), and CL in a chloroform/methanol mixture (4:1, v/v). A stock solution of the DPPG/POPG/CL mixture (1:1:2 mol/mol/mol) was then prepared at a final concentration of 1 mg/mL. All lipid monolayers were formed on a 0.01 M phosphate-buffered saline (PBS) solution (pH 7.4), either alone or in the presence of lipooligourea in the subphase. The lipid mixture was applied onto the subphase using a Hamilton syringe (50 μL). After spreading, the solutions were left undisturbed for 10 min to allow for complete solvent evaporation. Monolayers were compressed at a barrier speed of 10 mm/min under a constant temperature of 21 ± 1 °C, and surface pressure versus molecular area isotherms were recorded. To ensure reproducibility, all measurements were performed at least three times.

### Preparation of Unilamellar Vesicles

Stock solutions of the desired lipids (∼5.0 mg/mL) were prepared using the same method as for surface pressure measurements. The lipid solutions were then combined in a test tube at the required molar ratio (DPPG/POPG/CL 1:1:2). To remove the solvent, the mixture was vortexed under a nitrogen stream, and the resulting dried lipid film was further dried in a vacuum desiccator for 1 h to eliminate any residual solvent. Next, 1.0 mL of a 0.01 M phosphate-buffered saline (PBS) solution was added to the dried lipid film, and the mixture was bath sonicated at approximately 40 °C for 1 h. Following sonication, the lipid vesicle suspension appeared homogeneous and transparent. Vesicle size distribution was verified by dynamic light scattering (DLS), revealing diameters between 30 and 300 nm, with the distribution maximum at approximately 90 nm (see Supporting Information).

### Quartz Crystal Microbalance

Real-time measurements of mass and viscoelasticity changes in supported lipid bilayers upon exposure to lipooligourea were performed using a Q-Sense E4 quartz crystal microbalance with dissipation monitoring system (Q-Sense AB, Sweden). Silicon dioxide quartz sensor crystals (5 MHz, AT-cut) were obtained from the manufacturer and cleaned by sequential rinsing with Milli-Q water, followed by 30 min of sonication in 2% Hellmanex at 35 °C. After a final rinse with ultrapure water, the crystals were dried under UV lamp for 30 min and mounted into the flow module. To establish a stable baseline, phosphate-buffered saline (PBS) was circulated through the measurement chamber using a peristaltic pump at a flow rate of 0.20 mL/min for approximately 30 min. Solid-supported lipid bilayers were subsequently formed by injecting a suspension of unilamellar vesicles into the chamber at 0.15 mL/min (see Supporting Information file for details). Once a stable bilayer signal was confirmed, a solution of C10-OU4 in PBS was introduced at the same flow rate for 20 min. Flow was then halted, and the sample was incubated in the stagnant peptide solution for an additional 2 h. Final rinsing with buffer was performed to remove unbound molecules. Frequency (Δ*f*) and energy dissipation (Δ*D*) values were recorded at the fundamental frequency (5 MHz) and its first five overtones. For data interpretation, only overtone signals (n = 3 to 11) were considered, as the fundamental frequency is particularly susceptible to bulk solution effects and may yield misleading results.

### Attenuated Total Reflection-Fourier Transform Infrared Spectroscopy

All infrared spectra were acquired using a Nicolet iS50 spectrometer (Thermo Fisher Scientific Inc.) equipped with a liquid nitrogen-cooled MCT-A detector and a custom-built single-reflection accessory. The incident angle was set to 55°, and the spectral resolution was maintained at 4 cm^–1^. Spectra are presented in absorbance units, defined as A = log­(*I*_0_/*I*), where *I*_0_ and *I* represent the single-beam intensities of infrared radiation for the reference and the sample, respectively. A silicon hemispherical prism was used in all experiments. The prism was initially polished using polishing cloths with diamond suspensions of 3, 1, and 0.25 μm, with thorough rinsing in deionized water after each step. Subsequently, the prism was sonicated in 96% ethanol for 30 min. Following this cleaning procedure, the substrate was etched with a 40% NH_4_F solution for 2 min, followed by extensive rinsing with deionized water and methanol. Surface activation was started by applying a freshly prepared 1:1 (v/v) CH_3_OH/HCl solution to the prism and left for 30 min. The substrate was then rinsed with methanol and water. The prism was immersed in a 5% (v/v) APTES solution in ethanol for 20 min. Subsequently, it was rinsed with a 6% (v/v) solution of CH_3_COOH in methanol. After an initial rinse, the prism was sonicated in the same solution for 5 min, followed by thorough rinsing with methanol and water and air-drying. Before the lipid solution was applied, the background spectrum was recorded in 1 mL of deuterated PBS (*I*_0_). The prism was then rinsed with methanol and chloroform to remove the rest of the water solution, and left to dry. After that, 1 mL of a 3 mg mL^–1^ lipid solution was added on top of the prism and incubated for 15 min to initiate rapid solvent exchange procedure.^26–28^ Solvent exchange was initiated by the rapid addition of 1 mL of deuterated PBS, which resulted in the formation of a cloudy and foamy mixture. Subsequently, 1 mL of the supernatant was removed and discarded. This procedure was repeated until the solution above the prism became clear, indicating the complete removal of chloroform. Finally, the cell contained 1 mL of deuterated PBS. Refractive indices applied in molecular orientation calculations were 3.42 for silicon, 1.45 for the lipid bilayer, and 1.32 for D_2_O. Data analysis was conducted using Omnic 9 software (Thermo Fisher Scientific Inc.).

## Results and Discussion

The influence of C10-OU4 lipooligourea on model bacterial lipid membranes was investigated using the Langmuir technique. As a representative membrane system, we employed a negatively charged lipid monolayer composed of DPPG, POPG, and cardiolipin in a 1:1:2 ratio.^24^ The lipooligourea was introduced into the aqueous subphase containing 0.01 M PBS at a final concentration of 1 μM. The obtained results are illustrated in Figure 1. Initially, the lipid monolayers were compressed on a PBS subphase in the absence of lipooligourea. The surface pressure (Π) vs molecular area (*A*) isotherms for the DPPG/POPG/CL monolayer exhibit a lift-off point at approximately 175 Å^2^. Upon reaching a surface pressure of around 20 mN/m, a phase transition from the liquid-expanded to the liquid-condensed phase is observed. Partial monolayer collapse occurs at ∼42 mN/m, corresponding to the expulsion of POPG molecules from the monolayer, as reported in previous studies.^29^ The removal of POPG leads to increased condensation of the monolayer, with the remaining DPPG and cardiolipin components undergoing further compression until a second collapse event occurs at ∼ 68 mN/m. Since the primary objective of this study was to assess the impact of lipooligourea on a three-component monolayer, data collected beyond the collapse of POPG were excluded from further analysis. Introducing lipooligourea into the subphase results in a shift of the DPPG/POPG/CL isotherm toward larger molecular areas. This shift suggests that lipooligourea molecules integrate into the monolayer, with the effect being most pronounced in the early stages of compression. The lift-off point of isotherms recorded in the presence of C10-OU4 in the subphase occurs at approximately 420 Å^2^, indicating that at low surface pressure, lipooligourea efficiently incorporates into the DPPG/POPG/CL membrane.

**Figure 1:**
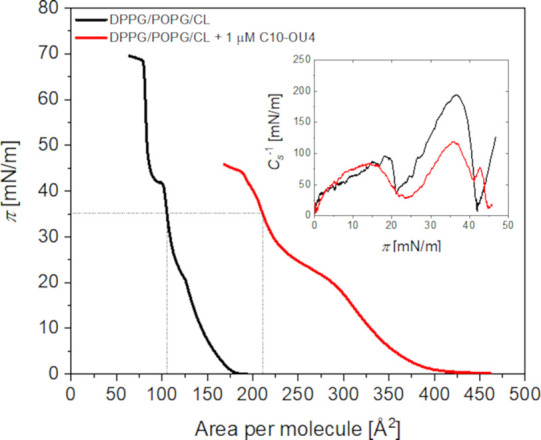
Surface pressure (Π) vs area per molecule isotherms for DPPG/POPG/CL monolayers recorded in the absence (black curves) and presence (red curve) of lipooligourea C10-OU4 (1 μM) dissolved in the subphase. The subphase consisted of an aqueous 0.01 M PBS solution. The insets depict the changes in the compression modulus as a function of surface pressure.

A more quantitative assessment of lipooligourea incorporation into lipid monolayers can be achieved by comparing molecular area values at a defined surface pressure. Specifically, at 35 mN/m, the molecular arrangement within the monolayer closely resembles that of natural cell membranes. In the absence of lipooligourea, the molecular area of the lipid membrane is approximately 105 Å^2^. However, in the presence of C10-OU4, this value increases to ∼ 210 Å^2^, indicating a substantial ability of lipooligourea to integrate into the monolayer during compression. Further insight into the properties of lipid monolayers can be obtained by evaluating the compression modulus (*C*_s_^–1^), which is described by the following equation:^30^

$$C_{s}^{-1}=-A\frac{d\Pi }{dA}$$ 1

where Π represents the surface pressure and *A* is the molecular area. This parameter provides valuable information about the physical state of the monolayer at a given surface pressure. It is generally accepted that a compression modulus in the range of 12.5–100 mN/m corresponds to a liquid-expanded state, 100–250 mN/m is indicative of a liquid-condensed state, while values exceeding 250 mN/m characterize a solid state. For the DPPG/POPG/CL monolayer compressed on a pure buffer subphase, the maximum compression modulus reaches 194 mN/m, confirming that the monolayer exists in a liquid-condensed state (see inset in Figure 1). Upon the introduction of lipooligourea at a concentration of 1 μM, this value significantly decreases to 119 mN/m. This result suggests that while the monolayer remains in the liquid-condensed state, the incorporation of C10-OU4 reduces the packing density of the lipid molecules.

Under physiological conditions antimicrobial agents interact with fully formed cell membranes, unlike the previously described system, where lipids were compressed in the presence of lipooligourea. Therefore, we examined the effects of lipooligourea on preformed monolayers at the air-buffer interface. To achieve this, lipid monolayers were initially compressed to a surface pressure of 35 mN/m, a value selected to mimic the structural organization of natural cell membranes. Following compression, the barriers of the Langmuir trough were locked to maintain a constant area occupied by the lipid film. Subsequently, surface pressure changes were continuously monitored over time, both in the absence and presence of lipooligourea (see Figure 2). To introduce C10-OU4 into the system, a stock solution was injected into the aqueous subphase beneath the monolayer, ensuring a final concentration of 1 μM.

**Figure 2:**
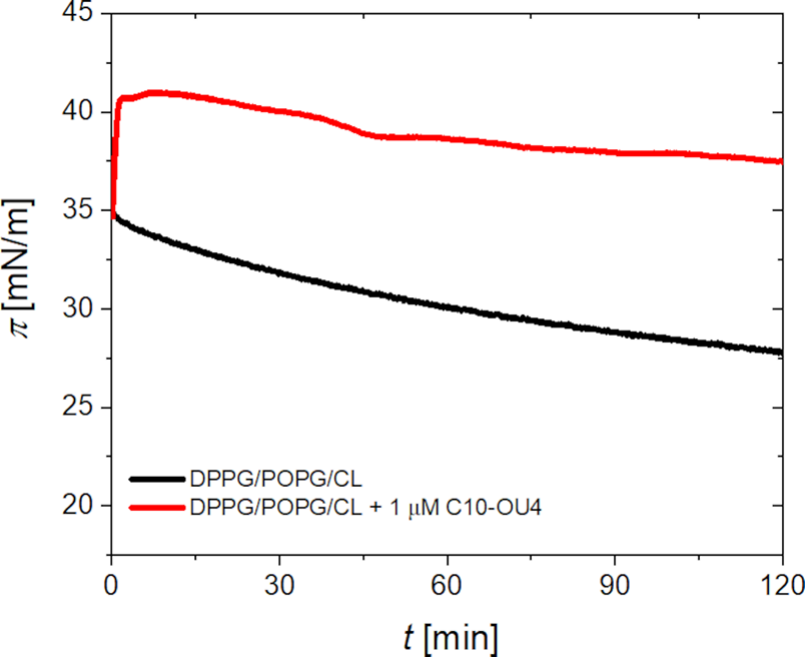
Time-dependent surface pressure changes of a DPPG/POPG/CL monolayer compressed to an initial pressure of 35 mN/m, under constant-area conditions at the air–water interface. The black curve represents the monolayer without any additional treatment, while the red curve corresponds to the monolayer after the injection of C10-OU4 into the subphase. Total concentration of C10-OU4 was 1 μM. The subphase consisted of an aqueous 0.01 M PBS solution.

In the absence of lipooligourea, the surface pressure of the DPPG/POPG/CL monolayer shows a slight decrease over time. This may be due to partial solubility of some lipid molecules in the aqueous subphase. Moreover, since all used lipids are negatively charged, and the monolayer compressed to 35 mN/m is very well packed and ordered, electrostatic repulsion between the headgroups could slowly destabilize the layer, leading to desorption of some molecules into the bulk. After injecting the C10-OU4 compound into the subphase, we observe a rapid increase in surface pressure by around 5–6 mN/m. This initial response, when surface pressure rises quickly and then begins to slightly decrease can be interpreted as a initial binding event, which is well described using pseudo-first-order kinetics (see Supporting Information). The obtained rate constant (k = 0.024 s^–1^) falls within, or even exceeds, the efficiency of binding reported for plasticins and polymyxin B interacting with lipopolysaccharide monolayers.^31^ At longer times, however, the system behavior becomes more complicated and cannot be described anymore by simple pseudo-first-order model. This could be due to reversible desorption, reorientation of the molecules, or lateral lipid reorganization in the monolayer. Also, equilibrium between bound and free states in subphase may play a role. Overall, the surface pressure changes clearly suggest that C10-OU4 interacts electrostatically with the negatively charged lipid monolayer due to its cationic character. However, this interaction is likely not strongly assisted by hydrophobic insertion, instead, C10-OU4 appears to remain mostly associated with the polar headgroup region.

While Langmuir monolayers are widely recognized as model membrane systems, they do not fully replicate the bilayer structure of biological membranes. To gain deeper insights into the membranolytic properties of lipooligourea, further investigations were conducted using solid-supported lipid bilayers.^32,33^ These bilayers offer a more accurate representation of natural cell membranes. However, they also come with certain limitations, primarily due to interactions between lipid molecules and the underlying substrate, which can influence factors such as the hydration state of the polar head groups in the lower leaflet. This issue is often mitigated by employing hydrophilic substrates like mica, glass, quartz or metal surfaces premodified with hydrophilic molecules.^32,34–37^ To investigate how the lipooligourea C10-OU4 influences the structural integrity of lipid membranes supported on hydrophilic surface, quartz crystal microbalance with dissipation monitoring (QCM-D) was employed. This technique enables real-time tracking of mass and mechanical properties changes in thin films by measuring shifts in resonance frequency (Δ*f*) and energy dissipation (Δ*D*) of a quartz sensor.^34,35,38^ A negative shift in frequency indicates the accumulation of material on the sensor, whereas a positive shift reflects mass loss. Changes in dissipation are indicative of alterations in film viscoelasticity: an increase in Δ*D* corresponds to a softer, more flexible layer, while a decrease suggests enhanced rigidity. Moreover, analyzing the frequency response across different overtones provides depth-resolved information about how these changes are distributed throughout the film.^39^ Each overtone penetrates the film to a different extent acoustically—lower overtones probe regions closer to the bulk solution, while higher overtones are more sensitive to areas near the sensor surface. Therefore, variations in the QCM-D response across multiple overtones reveal heterogeneity within the film, whereas uniform shifts across all overtones imply homogeneous structural or mechanical changes. Figure 3 presents the response of a quartz sensor modified with a DPPG/POPG/CL lipid bilayer in the presence of the lipooligourea C10-OU4 at concentrations of 1, 5, and 10 μM. At the lowest concentration of the compound (Figure 3A and B), a slight decrease in the oscillation frequency of the sensor was observed, on the order of 2–3 Hz, indicating a minor increase in mass deposited on the sensor surface. Concurrently, a slight decrease in dissipation was also noted, corresponding to a minor stiffening of the membrane. In both cases, the observed changes were rather modest, suggesting that at a concentration of 1 μM, the lipooligourea has a limited effect on the lipid bilayer, likely resulting primarily from adsorption of C10-OU4 molecules onto the membrane surface. However, negligible differences between overtones suggest that the membrane may be affected across its entire thickness, indicating possible incorporation of the lipophilic chains into the bilayer. Nevertheless, the number of interacting molecules appears relatively low. Due to the limited impact of C10-OU4 on the lipid membrane at low concentrations, we proceeded to investigate the effect of the compound at higher concentrations - closer to the minimal inhibitory concentrations (MICs) reported for C10-OU4.^19^ Figure 3C and D shows the sensor response in the presence of the lipooligourea at 5 μM. In this case, addition of C10-OU4 initially caused a gentle drop in frequency, followed by a pronounced increase of several Hz. Simultaneously, a marked decrease in dissipation was recorded. Furthermore, the responses at different overtones diverged significantly, suggesting that the membrane is not uniformly affected across its thickness. The most prominent changes were observed for the third overtone, with progressively weaker responses at higher overtones. This may indicate that the outer region of the membrane - i.e., the part exposed to the bulk solution - is the most affected. The substantial increase in frequency clearly points to a loss of mass from the sensor surface. This result may be interpreted as an effect of the lipooligourea damaging and dispersing the membrane, possibly through the formation of mixed lipid/lipooligourea micelles, which are subsequently removed from the surface and diffuse into the bulk solution. As a result, the sensor surface becomes locally uncovered by the soft layer, leading to the observed decrease in measured dissipation.

**Figure 3:**
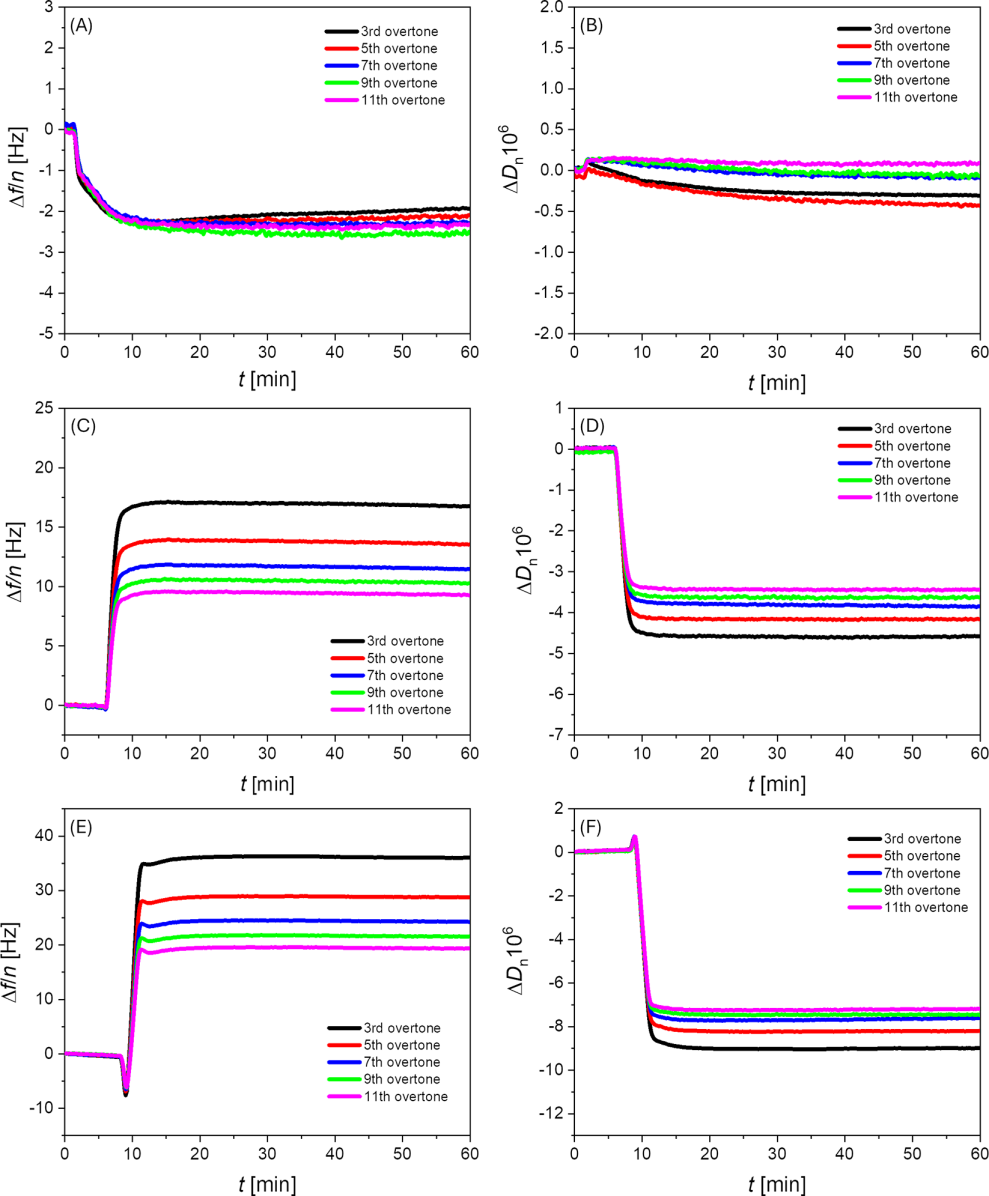
Representative QCM-D data showing changes in frequency (Δ*f*/*n*) and energy dissipation (Δ*D*) for quartz crystals coated with a DPPG/POPG/CL lipid bilayer upon exposure to the lipooligourea C10-OU4 at concentrations of 1 (A, B), 5 (C, D), and 10 μM (E, F). All measurements were conducted in 0.01 M phosphate-buffered saline (PBS) at room temperature.

A similar pattern of frequency and dissipation changes is observed at the higher concentration of the lipooligourea, which can be explained by the same underlying mechanism. However, immediately after the addition of C10-OU4 at a concentration of 10 μM, a distinct initial decrease in frequency is observed, accompanied by an increase in dissipation. This indicates accumulation of the active compound on the membrane surface. Shortly thereafter, the frequency increases sharply by 20–30 Hz, while dissipation simultaneously decreases, suggesting membrane micellization and removal of material from the sensor surface, in a manner analogous to the effect observed at 5 μM. In other words, higher concentrations comparable to the minimal inhibitory concentration (MIC) values lead to loss of membrane integrity and its subsequent dispersion.

Further insights into membrane alterations at the molecular level were investigated using ATR-FTIR spectroscopy, which allows quantitative insight into the structural organization of lipid bilayers before and after lipooligourea interaction. This method enables the evaluation of lipid orientation and molecular order within the membrane. Due to the polarization sensitivity of ATR-FTIR spectra, the technique is particularly well-suited for probing the alignment of lipid molecules adsorbed onto the planar surface of a silicon prism. The absorbance intensity of a given vibrational band depends on the angle between the transition dipole moment of that vibration and the electric field vector of the incident light. Accurate determination of molecular orientation therefore requires knowledge of both the direction and magnitude of the electric field at the interface, which can be precisely modulated using linearly polarized infrared radiation. When the evanescent wave penetrates significantly deeper than the thickness of the lipid film, the thin-film approximation becomes valid. Under such conditions, the spatial components of the electric field (*E*_x_, *E*_*y*_, *E*_*z*_) can be calculated using the following expressions:^40^

$$E_{x}=\frac{2\cos\theta_{1}{\left(\sin^{2}\theta_{1}-n_{31}^{2}\right)}^{1/2}}{{\left(1-n_{31}^{2}\right)}^{1/2}{\left[\left(1+n_{31}^{2}\right)\sin^{2}\theta_{1}-n_{31}^{2}\right]}^{1/2}}$$ 2

$$E_{y}=\frac{2\cos\theta_{1}}{{\left(1-n_{31}^{2}\right)}^{1/2}}$$ 3

$$E_{z}=\frac{2n_{32}^{2}\sin\theta_{1}\cos\theta_{1}}{{\left(1-n_{31}^{2}\right)}^{1/2}{\left[\left(1+n_{31}^{2}\right)\sin^{2}\theta_{1}-n_{31}^{2}\right]}^{1/2}}$$ 4

The angle of incidence of the infrared beam at the solid–liquid interface is denoted by θ_1_. Refractive index ratios are expressed as *n*_31_=*n*_3_/*n*_1_ and *n*_32_=*n*_3_/*n*_2_ where *n*_1_, *n*_2_, and *n*_3_ correspond to the refractive indices of the internal reflection element (silicon prism), the lipid bilayer, and the surrounding aqueous environment, respectively. In the case of a lipid membrane formed on the planar surface of a silicon prism, the dichroic ratio (*R*) can be experimentally determined by comparing the absorbance of p-polarized and s-polarized infrared light. With both the dichroic ratio and the evanescent field’s electric field amplitudes known, it is possible to calculate the orientational order parameter (*S*_dipole_) and the tilt angle (θ_dipole_) of a specific transition dipole moment with respect to the surface normal, using the equations:^41,42^

$$S_{dipole}=\frac{E_{x}^{2}-RE_{y}^{2}+E_{z}^{2}}{E_{x}^{2}-RE_{y}^{2}-2E_{z}^{2}}$$ 5

$$\theta_{dipole}=\cos^{-1}\sqrt{\frac{2S_{dipole}+1}{3}}$$ 6

When the molecular architecture of a film is well-characterized, a direct relationship can be established between the orientation of specific vibrational transition dipole moments and the molecular axis. For lipid molecules, the transition dipole moments associated with the symmetric and asymmetric CH_2_ stretching vibrations (ν_s_(CH_2_) and ν_as_(CH_2_)) are oriented perpendicular (α = 90°) to the molecular axis, which is defined by the trans-configured segments of the hydrocarbon chains. As a result, these vibrational modes serve as reliable indicators for estimating the average tilt angle of the acyl chains (θ_chain_) relative to the surface normal. Based on these measurements, the chain order parameter (*S*_chain_) can also be calculated, providing quantitative information on the conformational order and packing of the lipid acyl chains within the membrane. The value of the chain order parameter equals 1 when the hydrocarbon chains are fully aligned along the surface normal (i.e., parallel to the electric field vector), and drops to −0.5 when the chains are oriented perpendicular to the surface normal. In both scenarios, the hydrophobic regions of lipid molecules exhibit a well-defined orientation and limited conformational mobility. Conversely, an *S*_chain_ value of 0.0 corresponds to a completely random chain orientation, indicative of high dynamic freedom. Both parameters, *S*_chain_ and θ_chain_, can be calculated using following equations:

$$S_{chain}=\frac{E_{x}^{2}-RE_{y}^{2}+E_{z}^{2}}{\frac{1}{2}\left(3\cos^{2}\alpha -1\right)\left(E_{x}^{2}-RE_{y}^{2}-2E_{z}^{2}\right)}$$ 7

$$\theta_{chain}=\cos^{-1}\sqrt{\frac{2S_{chain}+1}{3}}$$ 8

In this study, the penetration depth of the evanescent wave at wavelengths corresponding to the C–H stretching region was estimated to be approximately 0.20 μm under the applied experimental conditions. Given that the lipid bilayer thickness is only ∼ 5.0 nm, the system meets the criteria for the thin film approximation, enabling accurate assessment of molecular orientation. The positions of the asymmetric ν_as_(CH_2_) and symmetric ν_s_(CH_2_) stretching bands of methylene groups provide insight into the physical state and packing density of acyl chains within lipid membranes. When the ν_as_(CH_2_) band appears below approximately 2920 cm^–1^ and the ν_s_(CH_2_) band falls below ∼ 2851 cm^–1^, this is indicative of a tightly packed, ordered gel phase, where the acyl chains are predominantly in an all-trans, fully extended conformation.^42,43^ In contrast, a shift of these bands to higher wavenumbers reflects increased conformational disorder due to the presence of gauche defects. In the fluid, liquid-crystalline phase, the ν_as_(CH_2_) and ν_s_(CH_2_) bands can reach up to ∼ 2924 cm^–1^ and ∼ 2853 cm^–1^, respectively. Figure 4 presents the ATR-FTIR spectra in the C–H stretching region for the DPPG/POPG/CL membrane deposited on the surface of a silicon prism, recorded in the absence (Figure 4A) and in the presence of C10-OU4 at concentrations of 1 μM (Figure 4B), 5 μM (Figure 4C), and 10 μM (Figure 4D), respectively. In the case of the intact membrane, the ν_as_(CH_2_) and ν_s_(CH_2_) bands are observed at 2918 cm^–1^ and 2851 cm^–1^, respectively, indicating that the acyl chains are in a well-ordered conformation and the membrane exists in the gel phase. By recording spectra using both p- and s-polarized light, the dichroic ratio was calculated to be ∼ 0.95–0.99, based on the intensities of ν_as_(CH_2_) and ν_s_(CH_2_) bands (see Table 1). From this, the order parameter of the acyl chains (*S*_chain_) was determined to be ∼ 0.72–0.80, further supporting the presence of a gel-like, highly ordered lipid phase. Additionally, the average tilt angle of the acyl chains relative to the membrane normal (θ_chain_) was found to be ∼ 24 ± 3° (based on the averaged values determined from ν_as_(CH_2_) and ν_s_(CH_2_) bands), which is consistent with values reported in our previous studies on the same membrane system.^25^ Interestingly, exposure of the lipid membrane to 1 μM C10-OU4 (Figure 4B) does not cause significant changes in the C–H stretching region of the spectrum. In practice, only a slight shift of the ν_as_(CH_2_) band toward higher frequencies is observed, reaching 2919 cm^–1^; however, the membrane still remains in the gel phase. Nevertheless, some alterations become apparent upon detailed analysis of the order parameter (*S*_chain_) and the tilt angle (θ_chain_), which are found to be in the range of 0.58–0.64 and 29–32°, respectively. Thus, the presence of the lipooligourea at a concentration of 1 μM induces minor changes in the orientation of the acyl chains and slightly reduces their ordering, but the overall physical state of the membrane remains unchanged. The situation is markedly different when considering the spectrum in Figure 4C, reflecting the effect of C10-OU4 at a concentration of 5 μM. In this case, a pronounced shift of both ν_as_(CH_2_) and ν_s_(CH_2_) bands to higher frequencies is observed—2925 cm^–1^ and 2854 cm^–1^, respectively. This indicates a significant increase in membrane disorder, which can be interpreted as enhanced fluidity. These spectral changes are clearly reflected in the *S*_chain_ value, which drops to ∼ 0.10–0.18. A reduction of this parameter toward zero unambiguously indicates a higher degree of acyl chain disorder and a greater contribution of random orientations. Furthermore, the corresponding tilt angle θ_chain_ reaches ∼ 48–51°, suggesting a substantial inclination of the acyl chains relative to the surface normal and a growing fraction of disordered lipid molecules. Similar conclusions can be drawn from the analysis of spectra recorded in the presence of C10-OU4 at a concentration of 10 μM (Figure 4D), since the *S*_chain_ and θ_chain_ parameters adopt similar values, i.e. ∼ 0.13–0.17 and ∼ 48–49°, respectively.

**Figure 4:**
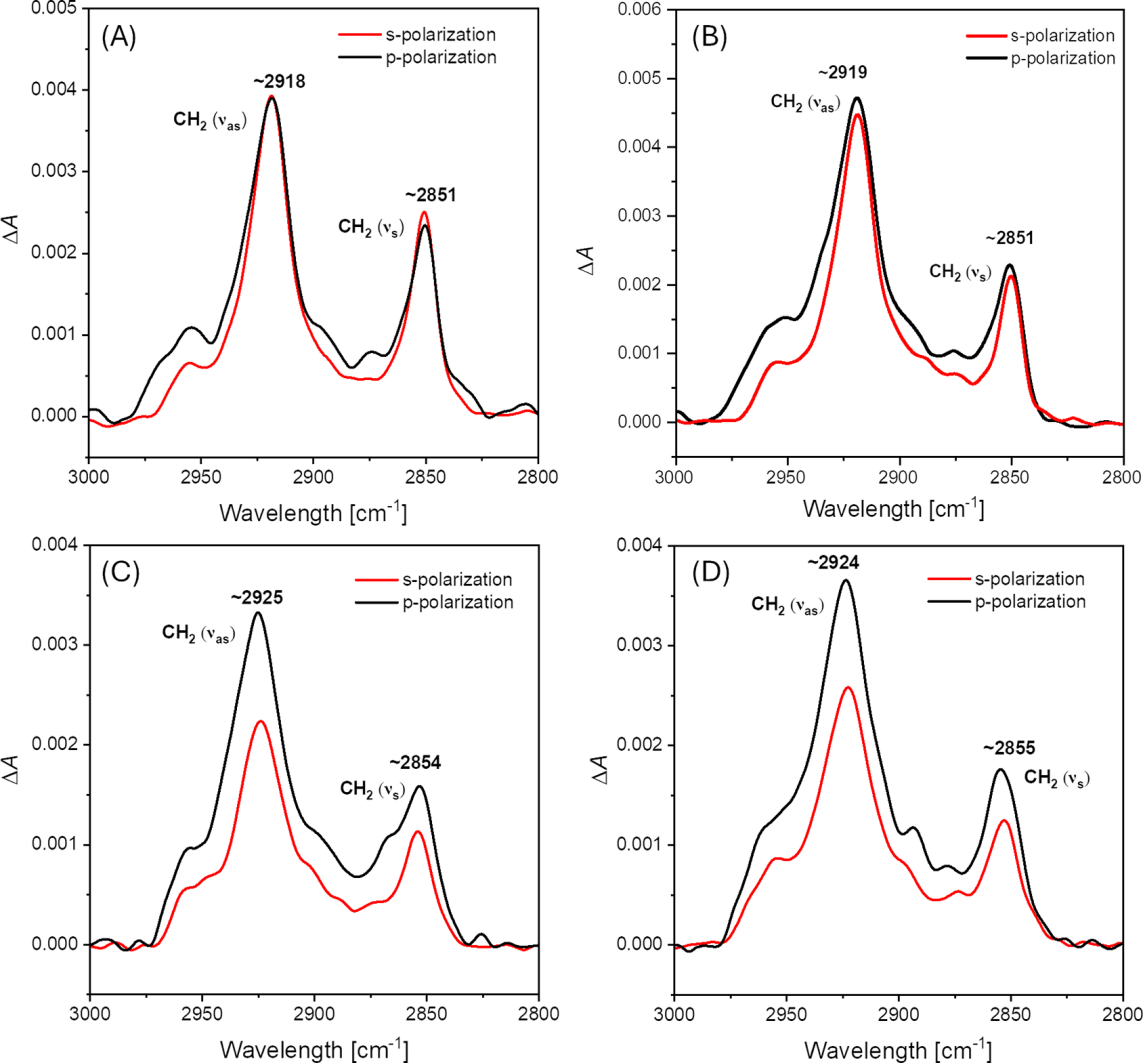
C–H stretching region of ATR-FTIR spectra recorded for DPPG/POPG/CL bilayer deposited onto the Si prism before (A) and after ∼60 min of exposure to 1 μM C10-OU4 (B); 5 μM C10-OU4 (C); and 10 μM C10-OU4 (D). The spectra were recorded in 0.01 M PBS dissolved in D_2_O. The red line corresponds to the spectra recorded with s-polarized light, while the black line corresponds to the spectra recorded with p-polarized light. A bare prism was used as a reference to assess global changes within hydrocarbon region.

**Table 1:** Molecular Ordering and Orientation Data

	dichroic ratio (*R*)		*S*_chain_		θ_**chain**_ **(deg)**	
lipid bilayer	**ν**_**as**_ **(CH**_**2**_ **)**	**ν**_**s**_ **(CH**_**2**_ **)**	**ν**_**as**_ **(CH**_**2**_ **)**	**ν**_**s**_ **(CH**_**2**_ **)**	**ν**_**as**_ **(CH**_**2**_ **)**	**ν**_**s**_ **(CH**_**2**_ **)**
DPPG/POPG/CL	0.993 ± 0.006	0.947 ± 0.006	0.717 ± 0.010	0.797 ± 0.010	25.7 ± 0.5	21.6 ± 0.6
DPPG/POPG/CL + C10-OU4(1 μM)	1.04 ± 0.02	1.08 ± 0.03	0.642 ± 0.032	0.581 ± 0.040	29.2 ± 1.4	31.9 ± 1.7
DPPG/POPG/CL + C10-OU4(5 μM)	1.47 ± 0.04	1.40 ± 0.02	0.105 ± 0.036	0.176 ± 0.022	50.6 ± 1.4	47.8 ± 0.8
DPPG/POPG/CL + C10-OU4(10 μM)	1.41 ± 0.01	1.44 ± 0.01	0.166 ± 0.011	0.135 ± 0.011	48.2 ± 0.4	49.4 ± 0.4

The ATR-FTIR results correlate well with the QCM-D measurements, where the effect of the lipooligourea at a low concentration (i.e., 1 μM) was limited to the binding/accumulation of a certain number of molecules within the membrane without compromising its integrity. Similar conclusions can be drawn from the ATR-FTIR data, which reveal only minor disturbances in the ordering of lipid acyl chains and a slight change in their tilt angle relative to the surface normal. According to the QCM-D results, at higher concentrations—namely 5 and 10 μM—significant membrane disruption occurs, and the bilayer is partially removed from the supporting surface. The ATR-FTIR findings support this scenario, as evidenced by a pronounced decrease in the acyl chain order parameter and a shift in the tilt angle toward values approaching the so-called magic angle. The magic angle (approximately 54.7°) is the specific orientation at which anisotropic interactions, such as those probed in IR spectroscopy, average out to zero. In this context, it indicates an increasingly disordered and randomly oriented population of acyl chains, consistent with membrane destabilization.

Further insight into the interaction of the lipooligourea with the lipid membrane can be obtained by analyzing the spectral region corresponding to the stretching vibrations of C = O and N–H bonds in the oligourea moiety of C10-OU4.^44^ Figure 5 presents the spectra in this region, where the characteristic urea I and urea II′ bands are visible. However, the urea II′ band is part of a complex envelope spanning the 1550–1400 cm^–1^ range, which comprises overlapping contributions from N–H/N–D bending modes resulting from isotopic exchange, as well as deformation vibrations of methylene groups and skeletal C–C vibrations originating from the aromatic phenyl rings. Although the urea II′ contribution—presumably dominated by N–D bending—appears prominent, definitive assignment of this band to a specific vibrational mode remains challenging due to the spectral complexity.

**Figure 5:**
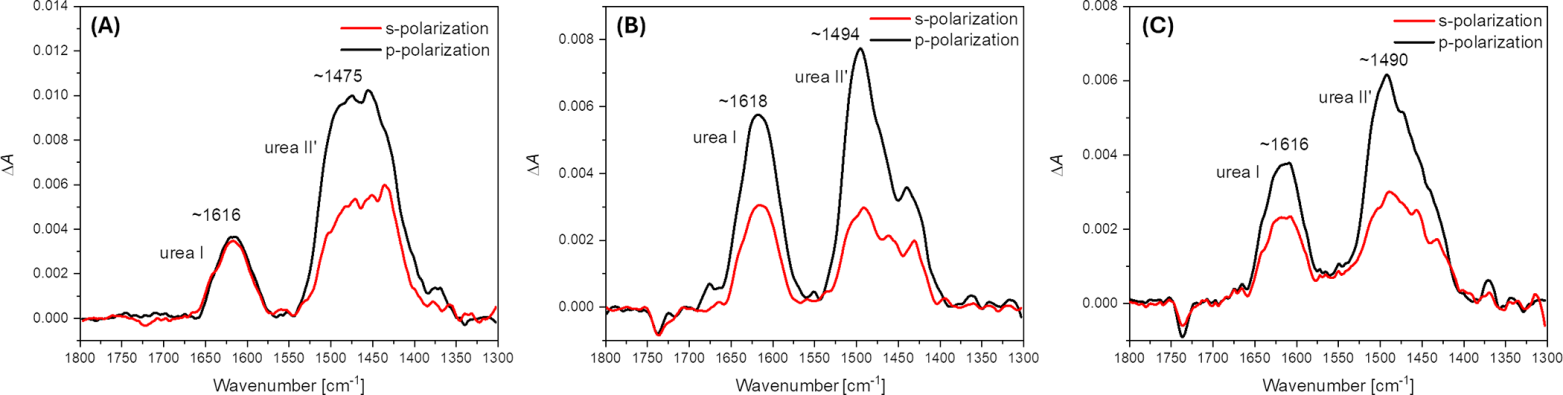
ATR-FTIR spectra in amide/urea region recorded for DPPG/POPG/CL bilayer deposited onto the Si prisms after ∼60 min of exposure to 1 (A), 5 (B), and 10 μM (C) C10-OU4. The spectra were recorded in 0.01 M PBS dissolved in D_2_O. The red line corresponds to the spectra recorded with s-polarized light, while the black line corresponds to the spectra recorded with p-polarized light. Spectra of intact membrane were used as a reference to assess the effect strictly related to lipooligourea action.

In contrast, the urea I band appears at higher wavenumbers, with a global maximum observed around 1615–1620 cm^–1^. Despite its complex nature, this band is generally attributed to the stretching vibrations of the carbonyl groups in the amide linkages. Its substantial bandwidth reflects structural heterogeneity within the helical segment of C10-OU4. Notably, the position of the urea I band is slightly red-shifted compared to typical values reported for a 2.5-helix conformation (commonly 1630–1640 cm^–1^).^45,46^ This shift toward lower frequencies suggests an enhancement in hydrogen bonding strength involving the amide groups, indicating potential additional stabilization of the helical structure. A similar effect has previously been observed for oligourea-based compounds in the presence of an electric field.^47^ However, in this case, the observed changes are more likely attributed to interactions with the polar headgroup region of the lipid membrane. Interestingly, no significant changes were observed in the position of the ester carbonyl stretching band of the lipids, which in our case remains quite weakly hydrated (band location at 1741 ± 1 cm^–1^). This implies that the ester group is either not directly involved in the interaction with the oligourea moiety, or its environment remains largely unaffected. Nevertheless, strong interactions with phosphate groups and/or glycerol residues in the polar headgroups of the lipids are plausible. Moreover, since the lipid headgroups are negatively charged while the oligourea moiety carries a positive charge, electrostatic interactions may contribute significantly to the observed stiffening of the oligourea helical structure.

As in the case of the C–H stretching region, a more quantitative analysis can also be attempted for the urea I region to estimate the instantaneous dipole moment angle of the C = O vibration relative to the surface normal. For C10-OU4 concentrations of 1 μM, 5 μM, and 10 μM, the corresponding θ_dipole_ values are 67.8 (± 1.9)°, 50.2 (± 0.2)°, and 55.1 (± 0.3)°, respectively. Assuming that the C = O groups are oriented approximately parallel to the helical axis, these values can be interpreted as the approximation of the tilt angle of the helix relative to the surface normal. This would imply that at the lowest concentration, the oligourea headgroup adopts an orientation more parallel to the membrane plane, whereas at higher concentrations, the helices assume a more vertical alignment. It must be emphasized, however, that this interpretation is a significant approximation due to the lack of detailed structural information regarding the conformation of the oligourea moiety. Moreover, the calculated angles are close to or even exceed the so-called magic angle (∼54.7°), which may suggest a random orientation of the oligourea headgroups. This scenario appears particularly plausible at higher concentrations, where micellization of the membrane is likely, in line with the QCM-D results. Nonetheless, it is evident that the behavior of C10-OU4 at the lowest concentration differs markedly from that observed at 5- and 10-fold higher concentrations. This distinction is further supported by the previously discussed QCM-D measurements, as well as the results of C–H stretching region and acyl chain ordering.

## Conclusions

This study elucidates the interaction mechanisms of the lipooligourea C10-OU4 with bacterial membrane mimetics using a combination of Langmuir monolayer measurements, QCM-D, and ATR-FTIR spectroscopy. The results indicate that the activity of C10-OU4 is highly concentration-dependent and modulated by both electrostatic and hydrophobic interactions with the membrane.

At low concentrations (1 μM), C10-OU4 shows modest interaction with lipid monolayers and bilayers, primarily incorporating into the polar headgroup region. The Langmuir isotherms as well as QCM-D suggest efficient surface adsorption, while ATR-FTIR reveal minor perturbations in acyl chain orientation and order. The compression modulus and the order parameter *S*_chain_ decrease slightly, but the membrane retains its gel-like structure. These findings suggest that the molecule predominantly aligns parallel to the membrane plane due to the electrostatic interactions between the positively charged oligourea moiety and the negatively charged phospholipid headgroups (e.g., cardiolipin, DPPG), possibly with lipophilic chains partially penetrating the hydrophobic core.

As the concentration increases to 5 μM, the mechanism of action shifts markedly. The QCM-D data indicate partial membrane solubilization, likely due to the formation of mixed lipid–lipooligourea micelles, followed by detachment from the surface. Correspondingly, ATR-FTIR measurements reveal a dramatic loss of lipid chain order (*S*_chain_ drops to ∼ 0.14), along with a significant tilt of the acyl chains (θ_chain_ ≈ 49°). The orientation of the urea carbonyl groups suggests that the helical axis of C10-OU4 begins to align more vertically, possibly facilitating deeper penetration of the lipophilic tail into the membrane core. This behavior mimics that of membrane-penetrating antimicrobial lipopeptides, such as daptomycin, which integrate into membranes upon oligomerization and calcium-dependent activation, inducing membrane thinning or pore formation.^23,48,49^

At 10 μM, near the reported MIC, the effects intensify. A pronounced increase in QCM-D frequency shift and dissipation change indicates extensive membrane disintegration, accompanied by a loss of bilayer integrity. The orientation of the helical oligourea headgroup approaches the magic angle, suggesting a randomized or tilted insertion geometry, consistent with the formation of lipid–peptide aggregates or toroidal defects. The findings are reminiscent of detergent-like mechanisms known for some antimicrobial peptides and lipopeptides, where increased concentrations lead to membrane solubilization.

Taken together, these results suggest a multimodal mechanism of membrane interaction by C10-OU4, characterized by surface binding, partial insertion, and concentration-dependent membrane disintegration. However, the precise molecular details of these interactions remain to be fully elucidated. Future investigations should incorporate electrochemical techniques to monitor changes in membrane permeability in real-time, as well as advanced microscopy methods capable of capturing the in situ morphology of lipid membranes. These complementary approaches will provide critical insights into the dynamic behavior of C10-OU4 at biologically relevant interfaces and guide its development as a synthetic membrane-active agent.

## Supplementary Material



## Notes

Dynamic light scattering data for lipid vesicle size distribution; AFM images and cross-sectional analysis of planar lipid membranes; QCM-D data for membrane formation; kinetic analysis and modeling of lipooligourea insertion into lipid monolayers (PDF)
