# Supplementary material for: Mechanistic Insights into Lipooligourea-Lipid Membrane Interactions
Source: J Phys Chem B. 2025 Jun 21;129(26):6517–27. doi: 10.1021/acs.jpcb.5c02112 (PMC12235613; doi:10.1021/acs.jpcb.5c02112)
Supplement: Supplementary file 1 [file jp5c02112_si_001.pdf]

SUPPORTING INFORMATION

Mechanistic Insights into Lipooligourea-Lipid Membrane Interactions

Kinga Burdach,[1] Zuzanna Reterska,[1] Arkadiusz Grempka,[1] Damian Dziubak,[1] Joanna Juhaniewicz-Dębińska,[1] Paulina Bachurska-Szpala,[2] Karolina Pulka-Ziach,[2] Sławomir Sęk[1,2*]

[1]*University of Warsaw, Faculty of Chemistry, Biological and Chemical Research Centre, Żwirki i Wigury 101, 02-089 Warsaw, Poland.*

[2]*University of Warsaw, Faculty of Chemistry, Pasteura 1, 02-093 Warsaw, Poland.*

*Corresponding author: slasek@chem.uw.edu.pl

1. Evaluation of the size distribution of lipid vesicles using dynamic light scattering

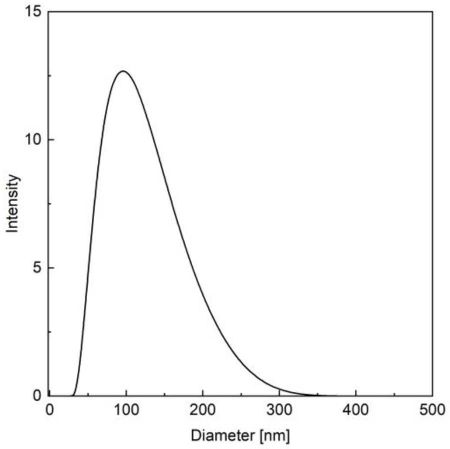

Figure S1. *Dynamic light scattering (DLS) results showing the size distribution of unilamellar lipid vesicles composed of DPPG/POPG/CL.*

2. **Evaluation of planar membrane formation on hydrophilic substrate by atomic force microscopy**

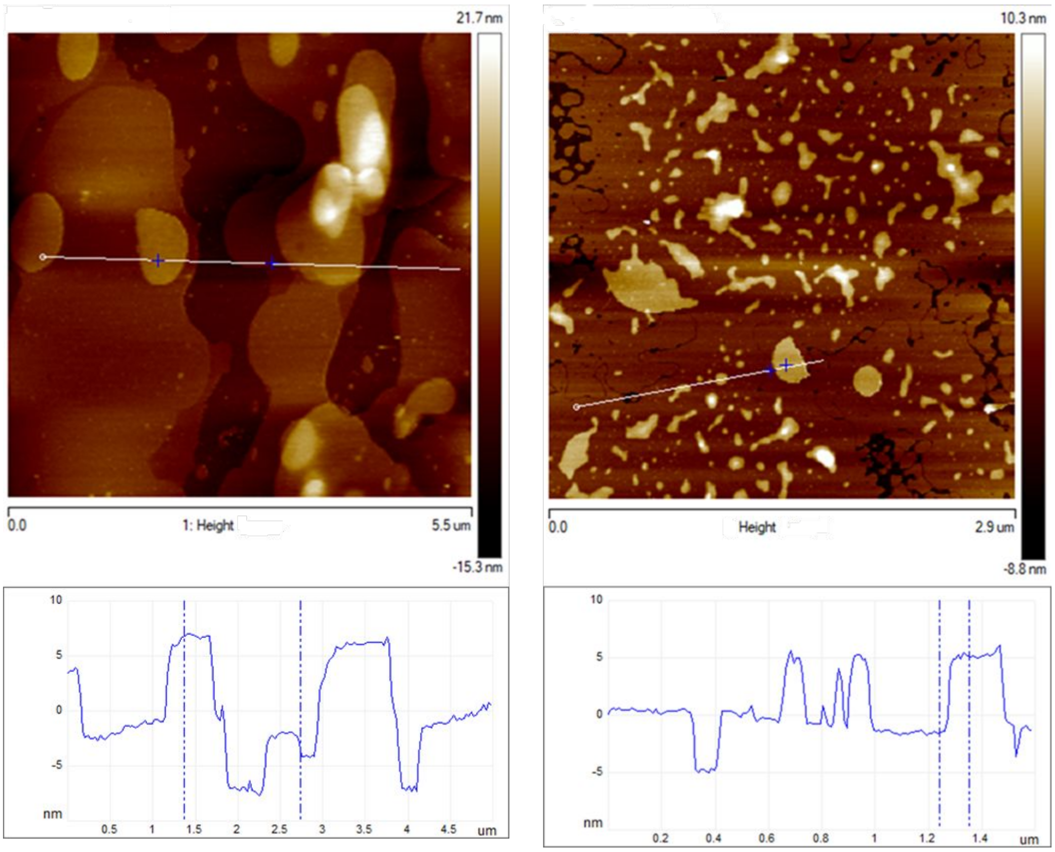

Figure S2. *Atomic force microscopy (AFM) images showing the formation of a planar lipid membrane from vesicles composed of DPPG/POPG/CL. The upper panels presents the topography images, revealing a continuous single lipid bilayer interspersed with brighter regions corresponding to multilayer patches (mostly double bilayer). The lower panel shows the cross-sectional profile along the white lines in the topography images, clearly indicating height variations consistent with the coexistence of single and multilayer areas.*

Planar membrane formation was verified using AFM imaging. A representative images are presented in Figure S2. As can be seen, the membrane does not exhibit phase separation and is generally homogeneous – only minor morphological differences are visible locally at the boundaries of merging domains (see right image in Figure S2), where lipid molecules are probably less ordered, leading to local thinning of the membrane. A clearly visible feature of the membrane are the locally occurring brighter "islands," which, based on cross-sectional analysis, correspond to regions where a multilayer is present – in most cases, a double lipid bilayer. This membrane morphology explains the higher mass and dissipation variation observed in QCM-D experiments, which are greater than expected for a single lipid bilayer. However, this does not affect the observed effect of lipooligourea activity and its destructive action leading to membrane dispersion/ damage.

3. Evaluation of planar membrane formation on hydrophilic substrate by quartz crystal microbalance

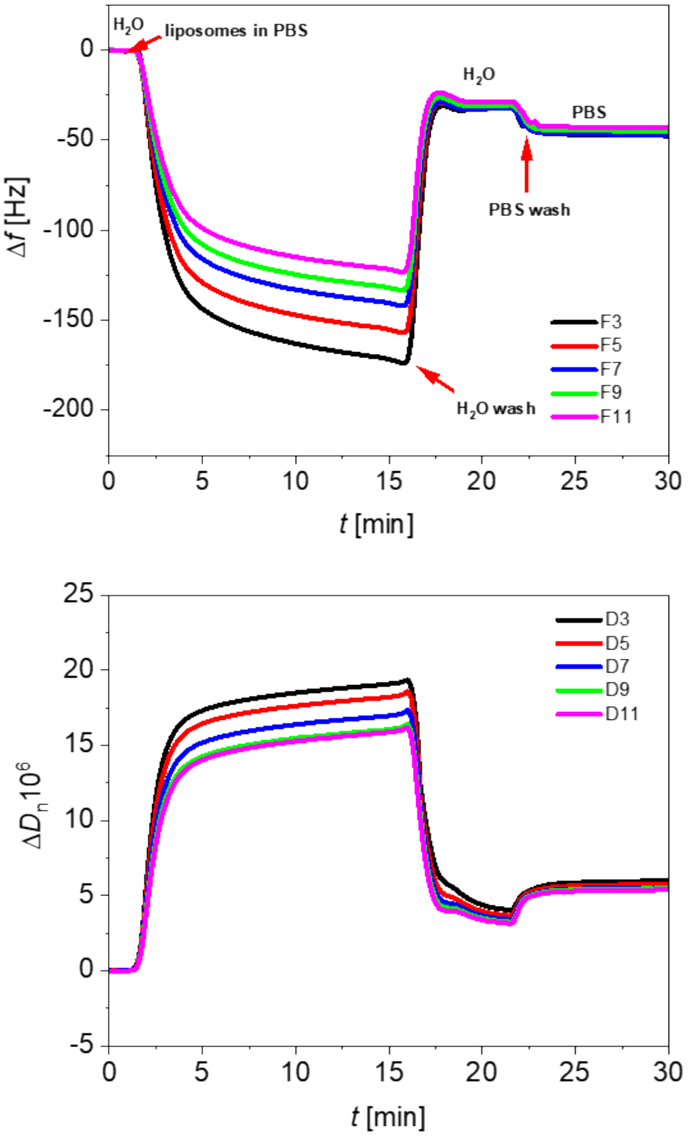

....

Figure S3. *Quartz crystal microbalance response during the formation of a planar lipid membrane from vesicles composed of DPPG/POPG/CL. The upper panels presents the changes in frequency, while the lower panel shows the changes in dissipation.*

The formation of the planar lipid membrane was also monitored using QCM-D (Figure S3). The initial pronounced decrease in frequency accompanied by an increase in dissipation indicates the adsorption of liposomes, which subsequently underwent rupture upon rinsing the system with pure water. This was followed by rinsing with PBS. The frequency then stabilized at approximately 40 Hz, suggesting the presence of multilayers, which is consistent with AFM

imaging. The dissipation level, higher than typically expected for a single lipid bilayer, further supports this interpretation. In other words, the applied procedure leads to the formation of a planar lipid membrane; however, locally it exists in a multilayered form—as indicated by AFM data, predominantly as a double bilayer structure.

4. Kinetics of lipooligourea insertion into the model lipid monolayer

To explore the kinetic behavior of C10-OU4 insertion into lipid monolayers, we analyzed the time-dependent surface pressure data shown in Figure 2 using a pseudo-first-order kinetic model, a standard approach in studies of amphiphilic compound adsorption at lipid interfaces.[1] This model is expressed as:

$$\Pi(t) = \Pi_0 + \left(\Pi_{eq} - \Pi_0\right)(1 - e^{\{-k\,t\}})$$

where:

- Π(t): surface pressure at time t,
- Π_0: initial surface pressure,
- Π_{eq}: equilibrium surface pressure,
- *k*: apparent first-order rate constant.

We fitted the model to the experimental data within time range of 0–20 minutes. The analysis yielded the following parameters:

- Equilibrium surface pressure Π_{eq} : 40.8 mN/m
- Apparent rate constant *k*: 0.024 s^{-1}

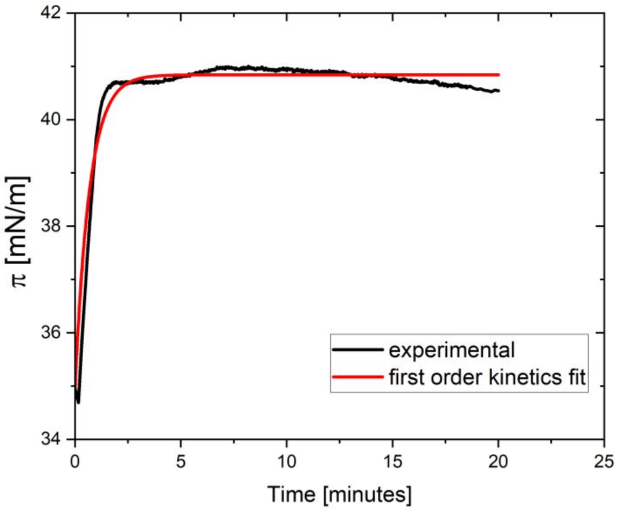

Figure S4. *Time-dependent increase in surface pressure following the insertion of C10-OU4 into a DPPG/POPG/CL lipid monolayer. Experimental data (black dots) were fitted with a pseudo-first-order kinetic model (red line). The model captures the initial adsorption phase.*

This result indicates that the early phase of C10-OU4 interaction with the monolayer is quite well-described by pseudo first-order kinetics, likely driven by electrostatic attraction and diffusion-controlled adsorption. However, while the pseudo first-order model provides a good approximation

over the initial time range, we note that its accuracy diminishes slightly at longer times, particularly beyond ~15 minutes. In this range, the pressure appears to approach a fluctuating pseudo-plateau, possibly due to:

- Reversible desorption or reorientation of the compound at the interface,
- Lateral lipid reorganization or structural relaxation within the monolayer,
- Weak dynamic equilibrium between surface-bound and subphase states.

These secondary processes are not captured by the single exponential model. For more detailed interpretation beyond the initial adsorption phase, more complex model might provide better results.

Literature:

(1) Michel, J. P.; Wang, Y. X.; Dé, E.; Fontaine, P.; Goldmann, M.; Rosilio, V. Charge and Aggregation Pattern Govern the Interaction of Plasticins with LPS Monolayers Mimicking the External Leaflet of the Outer Membrane of Gram-Negative Bacteria. *Biochimica et Biophysica Acta (BBA) - Biomembranes* **2015**, *1848* (11, Part A), 2967–2979. https://doi.org/https://doi.org/10.1016/j.bbamem.2015.09.005.
